# Supposed Precocious Menstruation

**Published:** 1897-05

**Authors:** 


### SUPPOSED PRECOCIOUS MENSTRUATION.

The reports of menstruation in infancy and childhood which are so often made are not to be too readily accepted. Comby (*L'Union Medicale*) found ordinary vulvo-vaginitis sometimes accompanied by distinct hemorrhages. In these cases of children, of two, three, and six years of age which had been mentioned as instances of precocious menstruation, he found that the blood came not from the uterus, nor from the vagina, but from vascular granulations about the meatus urinarius, which bled freely if touched. This condition was cured by weak injections of permanganate of potassium, and cauterization with a one in fifty solution of nitrate of silver.—*St. Louis Medical and Surgical Journal.*

